# Effect of Temporal Difference on Clinical Outcomes of Patients with Out-of-Hospital Cardiac Arrest: A Retrospective Study from an Urban City of Taiwan

**DOI:** 10.3390/ijerph182111020

**Published:** 2021-10-20

**Authors:** Han-Chun Huang, Tsung-Yu Lee, Cheng-Han Tsai, Yao-Sing Su, Yi-Rong Chen, Ya-Ni Yeh, Chi-Feng Hsu, Ming-Jen Tsai

**Affiliations:** 1 Department of Emergency Medicine, Ditmanson Medical Foundation Chia-Yi Christian Hospital, Chiayi City 600, Taiwan; lastar0329@gmail.com (H.-C.H.); dayna_dayna@hotmail.com (T.-Y.L.); masterdoctorsky@yahoo.com.tw (Y.-R.C.); cych13365@gmail.com (Y.-N.Y.); 2 Department of Emergency Medicine, Chiayi Branch, Taichung Veteran’s General Hospital, Chiayi City 600, Taiwan; chtsai6482@gmail.com; 3 Department of Information Management, Institute of Healthcare Information Management, National Chung Cheng University, Chiayi County 621, Taiwan; 4 Fire Bureau, Chiayi City Government, Chiayi City 600, Taiwan; fire001@ems.chiayi.gov.tw

**Keywords:** out-of-hospital cardiac arrest, temporal difference, circadian variation, nighttime, survival, return of spontaneous circulation

## Abstract

Circadian pattern influence on the incidence of out-of-hospital cardiac arrest (OHCA) has been demonstrated. However, the effect of temporal difference on the clinical outcomes of OHCA remains inconclusive. Therefore, we conducted a retrospective study in an urban city of Taiwan between January 2018 and December 2020 in order to investigate the relationship between temporal differences and the return of spontaneous circulation (ROSC), sustained (≥24 h) ROSC, and survival to discharge in patients with OHCA. Of the 842 patients with OHCA, 371 occurred in the daytime, 250 in the evening, and 221 at night. During nighttime, there was a decreased incidence of OHCA, but the outcomes of OHCA were significant poor compared to the incidents during the daytime and evening. After multivariate adjustment for influencing factors, OHCAs occurring at night were independently associated with lower probabilities of achieving sustained ROSC (aOR = 0.489, 95% CI: 0.285–0.840, *p* = 0.009) and survival to discharge (aOR = 0.147, 95% CI: 0.03–0.714, *p* = 0.017). Subgroup analyses revealed significant temporal differences in male patients, older adult patients, those with longer response times (≥5 min), and witnessed OHCA. The effects of temporal difference on the outcome of OHCA may be a result of physiological factors, underlying etiology of arrest, resuscitative efforts in prehospital and in-hospital stages, or a combination of factors.

## 1. Introduction

Out-of-hospital cardiac arrest (OHCA) is a universal public health problem that claims nearly 3.7 million lives annually [1]. Incidence and outcomes of OHCAs vary greatly around the world [2,3]. In a recent report from the International Liaison Committee on Resuscitation (ILCOR), the annual incidence of emergency medical services (EMS)-treated OHCA in a global population ranged from 30.0 to 97.1 per 100,000 individuals. Despite the advances in the treatment of OHCA, the rate of survival to discharge or 30-day survival remains poor at 3.1–20.4% across different regions of the world [2]. Increased understanding of the variables that affect OHCA clinical outcomes is important for developing preventative strategies and optimizing care for OHCA.

The Utstein Style guidelines consist of elements relevant to the clinical outcomes of OHCA. These include systemic factors (characteristics of the EMS system and the population served), dispatch factors (identified OHCA and dispatcher-assisted cardiopulmonary resuscitation (CPR)), patient factors (demographics, initial cardiac rhythm, witnessed status, arrest location, and bystander response), prehospital resuscitation process (response time, number of defibrillation shocks, quality of CPR, airway control, vascular access, delivery of epinephrine, etc.), and post-resuscitation processes (target temperature management, reperfusion therapy, etc.) [4]. It is important to understand how these core elements may be influenced by temporal differences.

There is significant circadian variation in the incidence of OHCA. The occurrence of OHCA is highest in the morning and lowest at night [5,6]. However, the association between temporal differences and clinical outcomes in patients with OHCA remains controversial [5,7,8,9,10,11]. Two studies conducted in Japan suggested that OHCA occurring at night had significantly lower rates of 30-day survival and survival with favorable neurological outcomes [7,8]. A similar finding from a USA study showed that the rate of survival to discharge was significantly lower for OHCA occurring at night than during the daytime [5]. On the other hand, two Austrian studies and one US/Canadian study observed no significant difference between OHCA occurring at daytime and nighttime with regard to the sustained return of spontaneous circulation (ROSC), survival to discharge, 30-day survival with favorable neurological outcome, and 1-year survival [9,10,11].

The cause of the relationship between temporal differences and varying clinical outcomes of OHCA remains unknown. In addition, there are no data concerning temporal differences and the clinical outcomes of OHCA in Taiwan. In this study, we aimed to evaluate the association between temporal differences and clinical outcomes of OHCA in an urban city in Taiwan; factors attributed to this observation were also evaluated in different OHCA statuses.

## 2. Materials and Methods

### 2.1. Study Design and Settings

A 3-year retrospective cohort study was conducted between 1 January 2018 and 31 December 2020 with the aim of evaluating temporal variations in clinical outcomes of OHCA in Chiayi City, Taiwan. Chiayi City extends across an area of 60.02 km^2^ and has 266,000 residents: It is the second-most densely populated city in Taiwan (4431.53 people per square kilometer) [12]. Residents aged over 65 years account for 16.2% of the Chiayi City population [12]. There are two primary hospitals, two secondary hospitals, and one tertiary referral hospital in the city. Patients with OHCA who require EMS are transferred to one of the five hospitals of the city. They are included in the OHCA registry of Chiayi City, a prospectively collected multi-center Utstein-style registry. Data were collected from the registry during the study period. Cardiac arrest, defined as the absence of signs of circulation, was verified by emergency medical technicians (EMTs) at the scene. Patients with traumatic cardiac arrest, including drowning and hanging, aged younger than 20 years, obvious death without being transferred to hospital, and those with valid do-not-resuscitate (DNR) orders were excluded from this study. The Institutional Review Board of the Ditmanson Medical Foundation Chia-Yi Christian Hospital approved this study (CYCH-IRB 2021057).

### 2.2. Emergency Medical Services System of Chiayi City

The EMS systems of Chiayi City have been described previously [12]. The fire-based EMS system of Chiayi City consists of seven ambulance stations and one dispatch center operated 24/7 by experienced EMTs. A total of 243 EMTs serve in the fire bureau, and the bureau is composed of 2 EMT-1 (0.82%), 212 (87.24%) EMT-2, and 29 (11.93%) EMT Paramedics (EMTP). Upon receiving a call for medical assistance, the dispatcher will ask key questions to identify patients with OHCA. EMTs are then dispatched from the nearest EMT stations as concurrent dispatcher-assisted CPR (DACPR) is conducted. At the rescue scene, EMTs provide basic life support, including CPR, ventilation by bag-valve-mask, insertion of a laryngeal mask airway, and automated external defibrillators (AED). If EMTPs are dispatched, advanced life support, including endotracheal intubation and epinephrine injection, can be provided. Unless ROSC is achieved, continued CPR, ventilation, and use of AEDs during transportation are mandated for all OHCA cases. All EMTs must receive regular CPR training according to national guidelines based on the American Heart Association, ILCOR, and European Resuscitation Council Guidelines. Quality assessments and controls are conducted monthly in order to ensure resuscitation quality.

### 2.3. Data Collection

Data were collected from the Chiayi City OHCA Registry. We obtained patient demographic information and recorded the time the EMS call was received (which was defined as the time of cardiac arrest); EMS response time (time from the call to the ambulance arriving at the scene); scene time (the time from the ambulance arriving at the scene to leaving the scene); transport time (the time from the ambulance leaving the scene to arriving at the hospital); total EMS time of identifying OHCA (defined as the sum of response time, scene time, and transport time); start time of DACPR or bystander CPR (BSCPR) from the time the call was received; number of dispatched EMTs; whether EMTPs were dispatched or not; location of cardiac arrest (public area, home, medical institution (local clinic and nursing home), ambulance transport, etc.); witness status of cardiac arrest; initial cardiac rhythm according to the AED record; prehospital management by EMTs; and the level of transferred hospital.

### 2.4. Patient Assignment and Outcome Measurement

**Figure 1:**
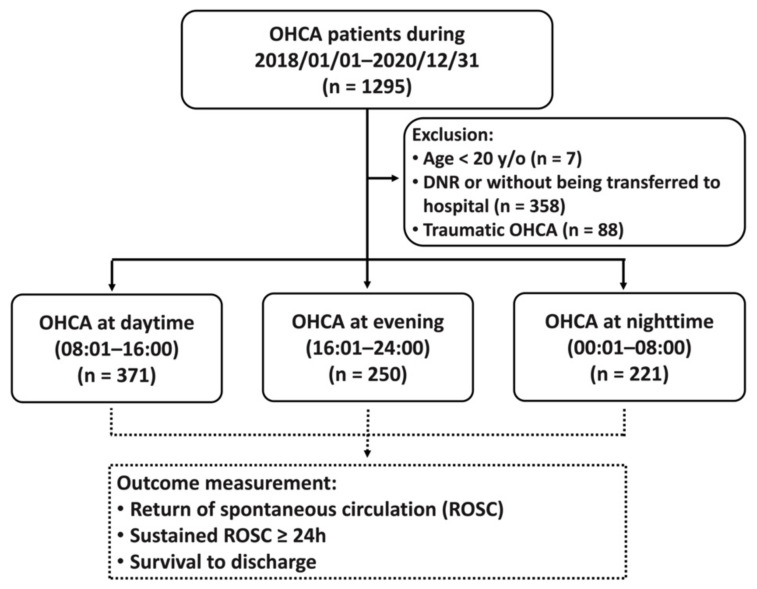
Flowchart of the patients included in the study. DNR: Do-Not-Resuscitate order; OHCA: out-of-hospital cardiac arrest.

In order to evaluate the association between circadian variation and clinical outcomes of OHCA, we divided the patients into three groups according to the time the EMS emergency call was received: daytime (08:01–16:00), evening (16:01–24:00), and night (00:01–08:00). The outcome measurement included achievement of ROSC at any time, sustained (≥24 h) ROSC, and survival to discharge (Figure 1).

### 2.5. Statistical Analysis

We hypothesized that 30% of OHCA occurred at nighttime, with an odds ratio of 0.6 in achieving ROSC in patients with an OHCA at nighttime compared to daytime, and set a ROSC rate of 30% as the baseline [5,7,13]. A two-tailed test size of 5% and a power of 80% was applied. We calculated that a total of 784 patients with OHCA would be required to reach statistical power. Therefore, we included three years of data to meet this requirement.

We analyzed and compared data of patients with an OHCA between the three groups: EMS call received during daytime (08:01–16:00), evening (16:01–24:00), and at night (00:01–08:00). For categorical variables, the chi-squared test was used to evaluate differences between groups. For continuous variables, analysis of variance (presented as mean ± standard deviation) or the Kruskal–Wallis analysis (presented as medians (interquartile range)) was used appropriately after evaluating the data distribution. In order to evaluate the net effect of temporal differences (daytime vs. evening vs. nighttime) on patient outcomes, logistic regression with forward selection Wald test was performed with adjustments for the associated predictors related to outcomes of OHCA and variables with a *p*-value < 0.1, as derived from the univariate analysis. The adjusted variables included age [14,15], sex [14,16], EMS time interval [17,18], dispatch of EMTP [19,20], DACPR or BSCPR [21,22,23,24], witnessed cardiac arrest [12], shockable initial rhythm [12,21], location of cardiac arrest [25], use of public AED before EMT arrival [16,26], pre-hospital injection of epinephrine [27,28], prehospital use of mechanical CPR devices [12,29], and level of transferred hospital [30]. Moreover, in order to evaluate the temporal differences in different types of OHCA, including different sex and age groups (younger or older than 65 years), different EMS response times (less or longer than 5 min), witnessed status, and shockable or non-shockable cardiac rhythm, subgroup analyses were also performed using logistic regression with adjustments for influencing factors. Statistical significance was set at *p* < 0.05. Statistical analysis was performed using IBM SPSS version 22 (IBM Corp., Armonk, NY, USA) statistical software packages.

## 3. Results

### 3.1. Baseline Characteristics and Clinical Outcome

During the study period, 1295 OHCA patients who had activated the EMS system were identified. Seven patients younger than 20 years old and 88 patients with traumatic cardiac arrest were excluded from the study, as well as 358 patients with DNRs or obvious death at the scene who were not transferred to the hospital. A total of 842 patients with OHCA were included in this study. There were 371 incidences of OHCA during the day (08:01–16:00), 250 OHCAs in the evening (16:01–24:00), and 221 OHCAs at night (00:01–08:00) (Figure 1). Significant differences in clinical outcomes were observed between the daytime, evening, and night temporal groups (Table 1). OHCAs that occurred at night had a significantly lower rate of achieving ROSC (19% vs. 28.57% vs. 26%, *p* = 0.033), sustained (≥24 h) ROSC (9.96% vs. 21.02% vs. 18.40%, *p* = 0.002), and survival to discharge (0.91% vs. 6.74% vs. 5.6%, *p* = 0.005) compared to OHCAs occurring in the daytime and evening, respectively (Table 1). For the OHCAs at night, EMS response time (*p* < 0.001), scene time (*p* = 0.001) and total EMS time (*p* < 0.001) were longer than those in OHCAs during daytime and evening. Additionally, the identification time of the OHCA by dispatcher was shorter (*p* = 0.001) and witnessed cardiac arrest was lower (*p* < 0.001) for the OHCAs at night. Moreover, the location of cardiac arrest was significantly different (*p* = 0.003). The majority of OHCAs occurred at home for all temporal groups; however, a higher percentage of daytime OHCAs occurred in public areas (10.24%) than nighttime, while nighttime OHCAs occurred more often in medical institutions (16.52%) (Table 1) than daytime OHCAs.

### 3.2. Temporal Difference and the Outcomes of OHCA

**Figure 2:**
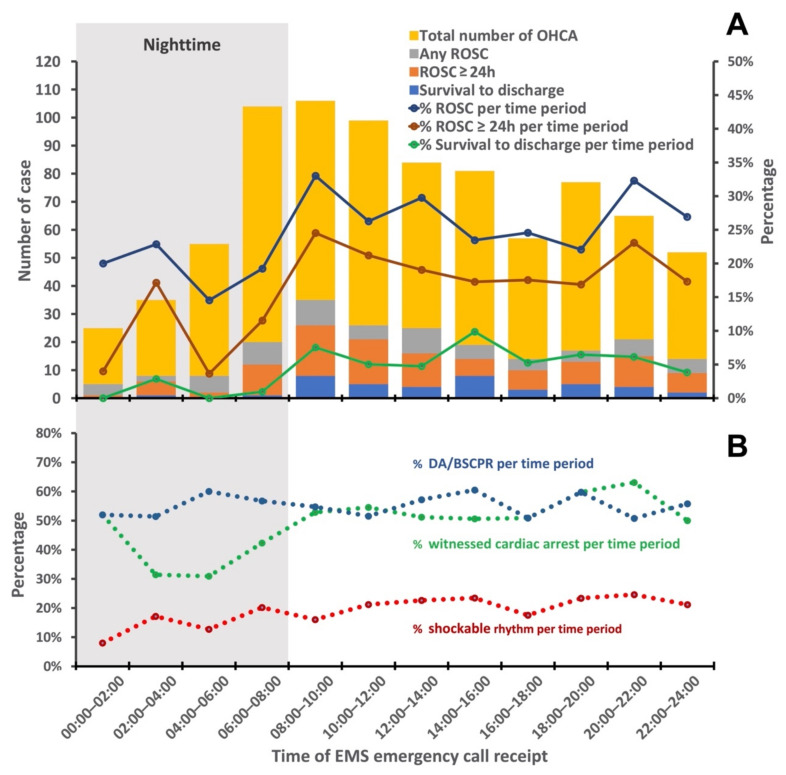
(**A**)Temporal variation of the frequency and percentage of OHCA, clinical outcomes, (**B**) DA/BSCPR, witnessed status, and initial cardiac arrest by 2 h intervals of a day. OHCA: out-of-hospital cardiac arrest; DA/BSCPR: dispatcher-assisted or bystander cardiopulmonary resuscitation.

Figure 2 shows how the frequency of OHCA incidents varies per 2-hourly intervals over 24 h as well as the percentage of achieving ROSC, sustained (≥24 h) ROSC, and survival to discharge. The rate of incidence of OHCA was higher during the daytime and peaked between 08:00 and 10:00. The rate of incidence decreased from daytime to evening, and a small peak was noted between 18:00 and 20:00. The lowest rate of incidence occurred at night between 00:00 and 04:00. The percentage per 2-hourly interval of achieving any ROSC, sustained (≥24 h) ROSC, and survival to discharge also showed temporal differences and were lower during the nighttime (00:00–08:00) (Figure 2A). In addition, the percentage of witnessed cardiac arrest per 2-hourly interval was lower between 02:00 and 06:00. However, no obvious temporal differences were found in the percentage of shockable rhythm and receiving DACPR or BSCPR (Figure 2B).

In order to assess the association of the occurrence time of OHCA with patient clinical outcomes and achievement of ROSC, multivariate analysis was performed. The variables with *p* values < 0.1 obtained from the univariate analysis (Table 1) and the associated factors related to outcomes of OHCA (described in the statistical analysis) were adjusted using logistic regression with forward selection analysis. The results showed that there was no significant difference between achieving ROSC in OHCA that occurred at night compared to OHCA that occurred during the daytime (adjusted odds ratio (aOR) = 0.74, 95% confidence interval (CI): 0.477–1.148, *p* = 0.18) (Table 2). The independent factors associated with achievement of ROSC were witnessed cardiac arrest (aOR = 2.362, 95% CI: 1.658–3.364, *p* < 0.001), occurrence of cardiac arrest in public areas (aOR = 3.666, 95% CI: 2.033–6.612, *p* < 0.001), ambulance transport (aOR = 3.944, 95% CI: 1.459–10.664, *p* = 0.007), use of public AED before EMT arrival (aOR = 3.421, 95% CI: 1.197–9.776, *p* = 0.022), and prehospital use of mechanical CPR device (aOR = 1.588, 95% CI: 1.122–2.248, *p* = 0.009) (Table 2).

The associations between the occurrence time of OHCA and achievement of sustained (≥24 h) ROSC and survival to discharge are shown in Table 3 and Table 4, respectively. In contrast to achieving ROSC, in the multivariate analysis, OHCA occurring at night had a significantly decreased probability of achieving sustained (≥24 h) ROSC (aOR = 0.489, 95% CI: 0.285–0.840, *p* = 0.009) compared to OHCA that occurred during the daytime (Table 3). The other independent factors associated with sustained (≥24 h) ROSC were similar to those of any ROSC, including witnessed cardiac arrest (aOR = 1.749, 95% CI: 1.159–2.64, *p =* 0.008), cardiac arrest occurring in the public area (aOR = 3.906, 95% CI: 2.096–7.28, *p <* 0.001), ambulance transport (aOR = 4.729, 95% CI: 1.654–13.52, *p* = 0.004), use of public AED before EMT arrival (aOR = 3.46, 95% CI: 1.218–9.826, *p =* 0.02), and prehospital use of mechanical CPR device (aOR = 1.761, 95% CI: 1.174–2.64, *p* = 0.006). Here, shockable rhythm was significant in this outcome (aOR = 1.557, 95% CI: 0.994–2.438, *p* = 0.053).

Consistent with sustained (≥24 h) ROSC, OHCA occurring at night also had a significantly decreased probability of achieving survival to discharge (aOR = 0.147, 95% CI: 0.03–0.714, *p* = 0.017) compared to OHCA occurring in the daytime (Table 4). Witnessed cardiac arrest (aOR = 2.85, 95% CI: 1.172–6.932, *p =* 0.021), shockable rhythm (aOR = 5.98, 95% CI: 2.868–12.469, *p* < 0.001), cardiac arrest in public areas (aOR = 3.523, 95% CI: 1.435–8.647, *p =* 0.006), and use of public AED before EMT arrival (aOR = 7.811, 95% CI: 1.873–32.581, *p =* 0.005) were independently associated with survival to discharge (Table 4).

### 3.3. Subgroup Analysis of the Temporal Difference on Different Characteristics of OHCA

**Figure 3:**
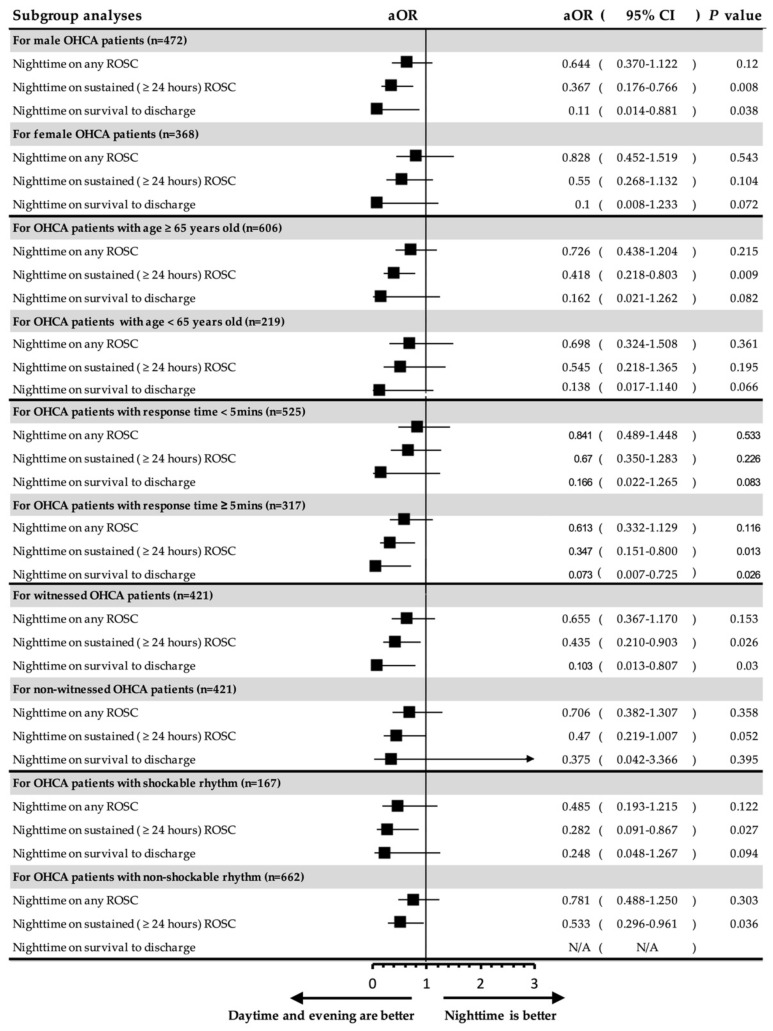
Subgroup analyses of nightmare (00:01–08:00) on the outcomes of OHCA patients. aOR: adjusted odds ratio; CI: confidence interval; OHCA: out-of-hospital cardiac arrest; ROSC: return to spontaneous circulation.

In order to analyze the association between the occurrence time of OHCA and clinical outcomes of different variables associated with OHCA, we conducted subgroup analyses according to sex, age group (≥65 years and <65 years), EMS response times (<5 min and ≥5 min), witness status, and initial cardiac rhythm. The confounding factors described above were also adjusted for. The results (Figure 3) revealed that male patients had a significantly decreased probability of achieving sustained ROSC (aOR = 0.367, 95% CI: 0.176–0.766, *p* = 0.008) and survival to discharge (aOR = 0.11, 95% CI: 0.014–0.881, *p* = 0.038) when OHCAs occurred at night compared to OHCAs that occurred in the daytime. However, this trend of temporal differences was not significant in female patients. Older adult patients (age ≥ 65 years) had a significantly decreased probability of achieving sustained ROSC (aOR = 0.418, 95% CI: 0.218–0.803, *p* = 0.009) when OHCA occurred at night, but this was not significant in younger patients (age < 65 years).

Significantly decreased probabilities of achieving sustained ROSC (aOR = 0.347, 95% CI: 0.151–0.8, *p =* 0.013) and survival to discharge (aOR = 0.073, 95% CI: 0.007–0.725, *p* = 0.026) were found at night, especially in OHCA patients with ≥ 5 min of EMS response time. However, there was no significant temporal difference in patients with < 5 min of EMS response time. With regard to witnessed cardiac arrest, decreased probabilities of achieving sustained ROSC (aOR = 0.435, 95% CI: 0.210–0.903, *p* = 0.026) and survival to discharge (aOR = 0.103, 95% CI: 0.013–0.807, *p* = 0.03) were also found at night, but these significances were not observed in non-witnessed OHCA. Regarding initial rhythm, both shockable rhythm (aOR = 0.282, 95% CI: 0.091–0.867, *p* = 0.027) and non-shockable rhythm (aOR = 0.533, 95% CI: 0.296–0.961, *p* = 0.036) showed significantly decreased probability of achieving sustained ROSC at nighttime than during daytime.

## 4. Discussion

In this study, we observed that the circadian pattern of the incidence of OHCA concurs with existing literature; that is, the incidence of OHCA is lower at night and peaks during the morning [6,31]. In addition, a temporal difference was found in the witness status of OHCA and the clinical outcomes of OHCA. After adjusting for the factors influencing survival, we found that there was no significant temporal difference in achieving ROSC (Table 2). However, the achievements of sustained (≥24 h) ROSC and survival to discharge were significantly poorer for incidences of OHCA at night (Table 3 and Table 4). Further subgroup analysis was performed, which found that temporal differences that influenced clinical outcomes were more significant, especially in male patients with older adult OHCA, OHCA with longer response time (≥5 min), and witnessed OHCA (Figure 3).

Core elements described in the Utstein-style guidelines are associated with clinical outcomes of OHCA [4]. However, temporal variations in these factors have not been thoroughly investigated. Regarding patient factors, our study showed a temporal difference in the witness status of OHCA and location of arrest. A lower percentage of witnessed cardiac arrest was found in nighttime incidences of OHCA compared to the daytime or evening (Table 1 and Figure 2). This indicates that a significant number of patients may experience an OHCA at night and die without being witnessed. Subsequently, the identification time of an OHCA by the dispatcher was shorter at night (Table 1). Additionally, a higher percentage of nighttime OHCA incidents occur in medical institutions and less in public areas than daytime or evening OHCAs. Notably, OHCAs occurring in public areas are known to have a better survival rate than in OHCAs occurring at home because of the increased probability of being witnessed and receiving BSCPR and AED [32,33]. Patients living in medical institutions (such as nursing homes) may have more comorbidities than those who do not live in medical institutions, which could contribute to the poor clinical outcomes of OHCA incidents at night. In order to control for these factors, a multivariate analysis was performed (Table 2, Table 3 and Table 4), which revealed that witnessed OHCAs and OHCA incidents in a public area were independently associated with clinical outcomes of OHCA. After adjusting for these confounding factors, achievements of sustained ROSC and survival to discharge were still significantly poor at night. The subgroup analysis specifically for witnessed arrest also showed consistent findings (Figure 3).

The association between patient demographics (age and sex) and temporal differences in the clinical outcomes of OHCA was examined, although no obvious differences were found in patient age and sex subgroups between daytime, evening, and nighttime (Table 1). We found that the temporal differences in clinical outcomes were more significant in male and older adult (≥65 years) patients in the subgroup analysis (Figure 3). The interactions between age and sex and the temporal difference may be explained by the underlying etiologies related to cardiac arrest. OHCAs of cardiac origin, such as acute myocardial infarction and subsequent ventricular arrhythmia, have a higher rate of incidence during the day compared to nighttime [34,35]. This implies that OHCA of non-cardiac origin may be more prevalent at nighttime compared to daytime. For males, the probability for one-month survival is lower among OHCA of non-cardiac origin than that of cardiac origin. This discrepancy was more significant in older adult men [14]. This observation may explain why the temporal difference in outcomes of OHCA was more significant in male and older adult patients.

Successful prehospital resuscitation can also be influenced by temporal differences, which further affects the outcomes of OHCA. According to previous literature, there are fewer personnel on duty during the night, which can disrupt the circadian rhythm of EMTs and potentially affect performance, motivation, and decision-making ability [36]. This study identified longer EMS response times and longer time spent at the rescue scene at night compared to in the daytime or evening, which may be due to personnel arrangement after midnight and that two EMTs were responsible for the medical calls from 00:00 to 08:00 a.m. Potential factors influencing response time include disruption of sleep and time required to get dressed. However, the number of dispatched EMTs, dispatch of EMTPs, and management during the prehospital phase (insertion of laryngeal mask airway, intravenous epinephrine injection, and use of mechanical CPR device) did not vary with time. Although slightly longer response times (median 5 vs. 4 min) and scene times (median 10 vs. 9 min) were found at night compared to the daytime and evening, there was no significant impact of these EMS time intervals on the clinical outcomes of OHCA in multivariate analysis (Table 2, Table 3 and Table 4). However, subgroup analysis (Figure 3) for OHCAs with a response time ≥ 5 min demonstrated that a poor outcome was more likely during the nighttime. Hence, the temporal group of EMS activity influences the clinical outcome of OHCA. [17,18,37] Conversely, this study has shown that OHCA incidents with relatively short response times and scene times did not have an impact on patient outcomes. Based on our finding, more resuscitation efforts may be needed for patients with prolonged response times, especially at night.

Previous studies investigating the association between temporal differences and clinical outcomes of patients with OHCA have shown inconsistent findings [5,7,8,9,10,11,38]. Studies by Matsumura et al. and Ho et al. both showed that 30-day survival was worse in patients with OHCAs occurring at night than during the day [7,38]. These two studies conducted in Asia both observed significantly lower applications of BSCPR during nighttime compared to daytime. Another study investigating the temporal variation in dispatcher-assisted and bystander-initiated resuscitation efforts also showed that the performance of DACPR or BSCPR was less prevalent at night compared to in the day [39]. The above studies attributed poor outcomes at night to lower dispatcher-assisted or bystander resuscitation efforts. Moreover, a recent study conducted in Vienna demonstrated no significant difference between day and night in sustained ROSC rates and survival with favorable neurological outcomes after OHCA, as nearly identical rates of BSCPR were found [9]. However, the relationship between DACPR and BSCPR, temporal differences, and the clinical outcomes of OHCA is still unclear. In contrast to previous studies, this study found no significant difference in the rates of DACPR/BSCPR and start time of DACPR (Table 1 and Figure 2). However, a significant association was found between temporal differences and clinical outcomes of OHCA. Thus, in addition to dispatcher-assisted or bystander resuscitation efforts, other factors contribute to poor outcomes at night.

Resuscitation efforts at the in-hospital stage for patients with OHCA have not been extensively studied. For in-hospital cardiac arrest (IHCA), similar temporal differences were observed. Incidences of IHCA during off-hours (at night and during the weekend) experience lower survival rates than incidences of IHCA during the daytime on weekdays [40]. Fewer in-hospital personnel resources during off-hours compared to on weekdays in the daytime may contribute to limited in-hospital resuscitation efforts and influence the patient clinical outcome [41,42]. A study by Matsumura et al. in Japan showed that resuscitation-associated procedures, such as endotracheal intubation and blood gas analysis, were performed less frequently at night compared to the day, despite no difference in the use of epinephrine and defibrillation during the nighttime [7]. One possible reason for this is that in-hospital care providers rarely work 24 h shifts in Japan [7,43]. A study conducted in Vienna investigated patients post-OHCA admitted to a specialized resuscitation center in which percutaneous coronary intervention is available 24/7, with a 30 min call-in-time at night. No differences in survival and neurologic outcomes between day and night admission were observed [11]. Hence, the temporal differences in the outcomes of OHCA may be affected by variation in the quality of post-resuscitation care. This study found no significant association between the temporal group and the initial achievement of ROSC (aOR = 0.74, *p* = 0.18) (Table 2). However, significance was exhibited in achieving sustained (≥24 h) ROSC (aOR = 0.489, *p =* 0.009) and even worse in achieving discharge survival (aOR = 0.147, *p =* 0.017). This may imply that resuscitation efforts from prehospital resuscitation to in-hospital resuscitation cannot be retained, especially at night. Although we adjusted the hospital factors according to the level of the transferred hospital, these findings still suggest that the quality of in-hospital resuscitation efforts may be inadequate at night. The concept of specialized post-arrest care centers for OHCA is needed.

This study has several limitations. First, as in other OHCA registry studies, the data regarding management during the in-hospital stage, such as the availability of post-resuscitation care, could not be sufficiently acquired and analyzed. Post-resuscitation care, such as revascularization therapy and therapeutic hypothermia, can influence the patient’s clinical outcome and may vary with the time of day. In order to account for this, adjustments were performed according to the level of the hospital where the patient with OHCA was sent in the study. Second, information on patients’ underlying diseases could not be obtained. Pre-existing conditions may have influenced the clinical outcomes of patients. However, this factor is unlikely to vary with time. Third, the time of EMS call received was used as a substitute for the time of cardiac arrest. This is not a precise measurement, especially for patients with unwitnessed cardiac arrest. However, it is a common problem in several studies [5,7,9,10,38].

## 5. Conclusions

Temporal differences influence the incidence and clinical outcomes of OHCA in Taiwan. Patients with OHCA at nighttime had a significantly decreased probability of achieving sustained ROSC and survival to discharge compared to patients with OHCA during the daytime and evening. This temporal difference was more significant in male patients, older adult patients, those with longer response times (≥5 min), and witness status. The temporal effects on the clinical outcome of OHCA may be a result of physiological factors, underlying etiology of arrest, resuscitative efforts in prehospital and in-hospital stages, or a combination of these factors. Further research into these findings may help to develop preventative strategies, improve ambulance deployment and hospital interventions, adjust resource allocation of EMS staff, and optimize care for OHCA.

## Figures and Tables

**Table 1:** Baseline characteristics and clinical outcomes by time of day of cardiac arrest occurrence.

	Time EMS Received Emergency Call									
	Daytime 08:01–16:00 (*n* = 371)			Evening 16:01–24:00 (*n* = 250)			Night 00:01–08:00 (*n* = 221)			*p* Value
Demographic characteristics										
Age (median)	77.0	(65–86)	(*n* = 363)	77.0	(62–86)	(*n* = 218)	77.0	(64–85)	(*n* = 218)	0.906
Age (mean)	73.2	(16.69)	(*n* = 363)	73.4	(16.02)	(*n* = 218)	72.8	(16.68)	(*n* = 218)	0.920
Older adults (≥65 years)	272	(74.93)	(*n* = 363)	173	(70.90)	(*n* = 244)	161	(73.85)	(*n* = 218)	0.538
Male sex	219	(59.03)		132	(52.82)		122	(55.20)		0.291
EMS time interval										
Response time (min)	4	(3–5)		4	(3–5)		5	(3–5)		<0.001
Scene time (min)	9	(7–11)		9	(7–12)		10	(8–13)		0.001
Transport time (min)	3	(2–4)		3	(2–4)		3	(2–4)		0.895
Total EMS time (min)	16	(14–19)		17	(14–19)		18	(15–22)		<0.001
EMS Dispatcher										
BSCPR or DACPR	207	(55.80)		136	(54.62)		124	(56.62)		0.907
Identification time of OHCA by dispatcher (sec)	60	(31–110)	(*n* = 272)	72	(31–125)	(*n* = 174)	43	(19–100)	(*n* = 163)	0.001
Start time of DACPR (sec)	168	(134–224)	(*n* = 188)	179	(141–235)	(*n* = 132)	175	(124–236)	(*n* = 109)	0.528
Number of dispatched EMT	3	(3–3)		3	(3–3)		3	(3–3)		0.201
Dispatch of EMTP	88	(23.72)		67	(26.80)		63	(28.51)		0.405
Characteristics of arrest										
Witnessed cardiac arrest	194	(52.29)		141	(56.40)		86	(38.91)		<0.001
Shockable initial rhythm	76	(20.82)		55	(22.45)		36	(16.44)		0.249
Location of cardiac arrest										
Home	282	(76.01)		202	(81.12)		157	(71.36)		0.003
Public area	38	(10.24)		11	(4.42)		10	(4.55)		
Medical institution	30	(8.09)		24	(9.64)		37	(16.82)		
Others	11	(2.97)		8	(3.21)		9	(4.09)		
During ambulance transport	10	(2.70)		4	(1.60)		7	(3.18)		
Prehospital treatment										
Laryngeal mask airway	310	(83.56)		210	(84.00)		181	(81.90)		0.813
Intravenous fluid injection	21	(5.68)		18	(7.20)		13	(5.88)		0.725
Intravenous epinephrine	19	(5.12)		16	(6.40)		12	(5.43)		0.788
Use of public AED before EMT arrival	9	(2.43)		6	(2.40)		6	(2.72)		0.970
Total number of AED defibrillations	0	(0–0)		0	(0–0)		0	(0–0)		0.065
Use of mechanical CPR device	166	(44.74)		104	(41.60)		101	(45.70)		0.629
Hospital level										
Primary hospital	59	(16.12)		26	(10.57)		34	(15.60)		0.095
Secondary hospital	190	(51.91)		137	(55.69)		99	(45.41)		
Tertiary hospital	117	(31.97)		83	(33.74)		85	(38.99)		
Outcomes										
Any ROSC	106	(28.57)		65	(26.00)		42	(19.00)		0.033
Sustained (≥24 h) ROSC	78	(21.02)		46	(18.40)		33	(9.96)		0.002
Survival to discharge	25	(6.74)		14	(5.60)		2	(0.91)		0.005

Values shown are *n* (%), mean (±SD), or median (interquartile range). AED: automated external defibrillators; BSCPR: bystander cardiopulmonary resuscitation (CPR); DACPR: dispatcher-assisted CPR; EMS: emergency medical services; EMT: emergency medical technician; EMTP: emergency medical technician paramedics; OHCA: out-of-hospital cardiac arrest; ROSC: return to spontaneous circulation.

**Table 2:** Predictors of achievement of any return of spontaneous circulation.

Variables	OR	(95% CI)	*p*-Value	aOR	(95% CI)	*p*-Value
Age (per year)	0.987	(0.978–0.997)	0.007		-	
Male sex	1.034	(0.756–1.415)	0.834		-	
Response time (per minute)	0.943	(0.867–1.026)	0.176		-	
Scene time (per minute)	0.967	(0.931–1.003)	0.071		-	
Dispatch of EMTP	0.94	(0.789–1.119)	0.486		-	
BSCPR or DACPR	0.795	(0.582–1.087)	0.15		-	
Shockable initial rhythm	1.865	(1.293–2.688)	<0.001		-	
Witnessed cardiac arrest	2.652	(1.912–3.679)	<0.001	2.362	(1.658–3.364)	<0.001
Location of cardiac arrest						
Home	reference			reference		
Public area	4.46	(2.582–7.705)	<0.001	3.666	(2.033–6.612)	<0.001
Medical institution	0.75	(0.424–1.327)	0.323	0.702	(0.379–1.301)	0.261
Others	1.665	(0.737–3.759)	0.22	1.904	(0.811–4.468)	0.139
During ambulance transport	4.685	(1.935–11.343)	<0.001	3.944	(1.459–10.664)	0.007
Use of public AED before EMT arrival	3.371	(1.411–8.054)	0.006	3.421	(1.197–9.776)	0.022
Prehospital epinephrine injection	1.413	(0.750–2.664)	0.285		-	
Total number of AED defibrillations	1.42	(1.167–1.728)	<0.001		-	
Use of mechanical CPR device	1.294	(0.947–1.767)	0.106	1.588	(1.122–2.248)	0.009
Hospital level						
Primary hospital	reference					
Secondary hospital	0.727	(0.460–1.150)	0.173		-	
Tertiary hospital	0.959	(0.596–1.541)	0.862		-	
Time of EMS call received						
Daytime (08:01–16:00)	reference			reference		
Evening (16:01–24:00)	0.878	(0.612–1.261)	0.482	0.989	(0.667–1.468)	0.957
Night (00:01–08:00)	0.587	(0.391–0.879)	0.01	0.74	(0.477–1.148)	0.18

OR: odds ratio; aOR: adjusted OR; BSCPR: bystander cardiopulmonary resuscitation (CPR); CI: confidence interval; DACPR: dispatcher-assisted CPR; EMS: emergency medical services; EMT: emergency medical technician; EMTP: emergency medical technician paramedics; OHCA: out-of-hospital cardiac arrest; ROSC: return to spontaneous circulation.

**Table 3:** Predictors of achievement of a sustained (≥24 h) return of spontaneous circulation.

Variables	OR	(95% CI)	*p*-Value	aOR	(95% CI)	*p*-Value
Age (per year)	0.987	(0.977–0.997)	0.012		-	
Male sex	0.903	(0.631–1.292)	0.577		-	
Response time (per minute)	0.885	(0.799–0.981)	0.02		-	
Scene time (per minute)	0.942	(0.899–0.986)	0.01		-	
Dispatch of EMTP	0.935	(0.765–1.141)	0.506		-	
BSCPR or DACPR	1.01	(0.704–1.448)	0.957		-	
Shockable initial rhythm	2.281	(1.529–3.404)	<0.001	1.557	(0.994–2.438)	0.053
Witnessed cardiac arrest	2.358	(1.619–3.434)	<0.001	1.749	(1.159–2.640)	0.008
Location of cardiac arrest						
Home	reference			reference		
Public area	5.302	(3.031–9.277)	<0.001	3.906	(2.096–7.280)	<0.001
Medical institution	1.143	(0.619–2.108)	0.67	1.15	(0.590–2.242)	0.681
Others	2.514	(1.074–5.882)	0.034	3.136	(1.275–7.712)	0.013
During ambulance transport	4.713	(1.930–11.512)	<0.001	4.729	(1.654–13.520)	0.004
Use of public AED before EMT arrival	4.579	(1.907–10.994)	<0.001	3.46	(1.218–9.826)	0.02
Prehospital epinephrine injection	0.977	(0.447–2.136)	0.953		-	
Total number of AED defibrillations	1.42	(1.155–1.746)	<0.001		-	
Use of mechanical CPR device	1.336	(0.934–1.909)	0.113	1.761	(1.174–2.640)	0.006
Hospital level						
Primary hospital	reference					
Secondary hospital	0.702	(0.420–1.171)	0.175		-	
Tertiary hospital	0.839	(0.492–1.431)	0.52		-	
Time of EMS call received						
Daytime (08:01–16:00)	reference			reference		
Evening (16:01–24:00)	0.847	(0.564–1.271)	0.423	0.963	(0.617–1.501)	0.867
Night (00:01–08:00)	0.415	(0.250–0.689)	<0.001	0.489	(0.285–0.840)	0.009

OR: odds ratio; aOR: adjusted OR; BSCPR: bystander cardiopulmonary resuscitation (CPR); CI: confidence interval; DACPR: dispatcher-assisted CPR; EMS: emergency medical services; EMT: emergency medical technician; EMTP: emergency medical technician paramedics; OHCA: out-of-hospital cardiac arrest; ROSC: return to spontaneous circulation.

**Table 4:** Predictors of achievement of survival to discharge.

Variables	OR	(95% CI)	*p*-Value	aOR	(95% CI)	*p*-Value
Age (per year)	0.971	(0.956–0.987)	<0.001		-	
Male sex	0.898	(0.479–1.685)	0.738		-	
Response time (per minute)	0.944	(0.793–1.122)	0.511		-	
Scene time (per minute)	0.935	(0.860–1.016)	0.113		-	
Dispatch of EMTP	0.935	(0.765–1.141)	0.506		-	
BSCPR or DACPR	1.019	(0.541–1.917)	0.954		-	
Shockable initial rhythm	7.954	(4.106–15.405)	<0.001	5.98	(2.868–12.469)	<0.001
Witnessed cardiac arrest	5.196	(2.277–11.857)	<0.001	2.85	(1.172–6.932)	0.021
Location of cardiac arrest						
Home	reference			reference		
Public area	7.952	(3.763–16.803)	<0.001	3.523	(1.435–8.647)	0.006
Medical institution	0.313	(0.042–2.348)	0.258	0.138	(0.014–1.384)	0.092
Others	2.164	(0.483–9.698)	0.313	1.852	(0.368–9.311)	0.455
During ambulance transport	4.689	(1.286–17.106)	0.019	3.329	(0.599–18.504)	0.169
Use of public AED before EMT arrival	8.983	(3.287–24.552)	<0.001	7.811	(1.873–32.581)	0.005
Prehospital epinephrine injection	0.862	(0.202–3.681)	0.841		-	
Total number of AED defibrillations	2.101	(1.615–2.735)	2.101		-	
Use of mechanical CPR device	0.993	(0.528–1.869)	0.983		-	
Hospital level						
Primary hospital	reference					
Secondary hospital	0.788	(0.325–1.911)	0.598		-	
Tertiary hospital	0.827	(0.325–2.102)	0.689		-	
Time of EMS call received						
Daytime (08:01–16:00)	reference			reference		
Evening (16:01–24:00)	0.821	(0.418–1.612)	0.567	0.988	(0.460–2.123)	0.975
Night (00:01–08:00)	0.126	(0.030–0.539)	0.005	0.147	(0.030–0.714)	0.017

OR: odds ratio; aOR: adjusted OR; BSCPR: bystander cardiopulmonary resuscitation (CPR); CI: confidence interval; DACPR: dispatcher-assisted CPR; EMS: emergency medical services; EMT: emergency medical technician; EMTP: emergency medical technician paramedics; OHCA: out-of-hospital cardiac arrest; ROSC: return to spontaneous circulation.

## Data Availability

The data that support the findings of this paper are available from the corresponding author, M.-J.T., upon reasonable request.
